# Efficacy of Probiotics as Adjunctive Therapy to Nonsurgical Treatment of Peri-Implant Mucositis: A Systematic Review and Meta-Analysis

**DOI:** 10.3389/fphar.2020.541752

**Published:** 2021-01-18

**Authors:** Rui Zhao, Huimin Hu, Yan Wang, Wenli Lai, Fan Jian

**Affiliations:** Department of Orthodontics, State Key Laboratory of Oral Diseases and National Clinical Research Center for Oral Diseases, West China Hospital of Stomatology, Sichuan University, Chengdu, China

**Keywords:** nonsurgical treatment, probiotic, systematic review, adjunctive treatment, peri-implant mucositis

## Abstract

**Background:** Peri-implant mucositis (PiM) is an inflammation of the soft tissues surrounding the dental implant and is the precursor of the destructive inflammatory peri-implantitis. PiM is usually reversible, but difficult to eradicate. Mechanical debridement (MD) is the conventional procedure to treat PiM although not enough to reach a complete resolution. Recently, probiotics have been considered in the treatment of peri-implant disease. Therefore, the aim of this systematic review and meta-analysis was to investigate the efficacy of the probiotic therapy combined with MD compared with MD alone or MD + placebo in patients with PiM.

**Methods:** A search using electronic databases (MEDLINE, Science Direct databases, and Cochrane Central Register of Controlled Trials) and a manual search were performed up to November 2019 by two reviewers independently of each other. Eligible randomized controlled trials (RCTs) comparing MD + probiotic vs. MD were included. The quality assessment for all the selected RCTs was conducted according to the Cochrane Handbook for Systematic Reviews of Interventions. Probing depth reduction was selected as the primary outcome. Weighted mean difference (WMD) and 95% confidence interval (CI) were calculated for continuous outcomes, and odds ratio (OR) and 95% CI were calculated for dichotomous outcomes, using random effect models. This review was registered on the PROSPERO database (CRD42020213625).

**Results:** Five eligible publications were included in this systematic review and four in the meta-analysis. As regards the implant, the WMD in the probing depth reduction between the test and control group was −0.12 mm [95% CI (−0.38, 0.14), *p* = 0.38], meaning that the adjunctive probiotic therapy was not improving PiM compared with MD alone or MD + placebo. The meta-analysis also showed no statistically significant results in the secondary outcomes (reduction of full mouth plaque index and full mouth bleeding on probing, absence of bleeding on probing at implant level, and changes in microorganism load and species).

**Conclusion:** The findings of this systematic review and meta-analysis suggested that the additional use of probiotics did not improve the efficacy of MD in PiM treatment regarding clinical and microbial outcomes, at least in a short-term.

## Introduction

Peri-implant mucositis (PiM) is a reversible inflammatory disease that occurs in the peri-implant soft tissues without alveolar bone loss, frequently found in patients with dental implants (Casado et al., 2019). Salvi et al. did a systematic review and reported a PiM prevalence of 43% (range: 19–65%) (Salvi et al., 2019). According to many experts, PiM is the precursor of the destructive inflammatory peri-implantitis and seems a sign of host response to the bacterial load (Heitz-Mayfield et al., 2012). Following reasonable treatment, PiM is fully reversible (Salvi et al., 2012). However, if improperly treated, the inflammation of the peri-implant soft tissues may lead to the irreversible bone loss of the implant-supporting structures, thus causing peri-implantitis (Costa et al., 2012). Since the success of the peri-implantitis treatment is limited it is difficult to eradicate and control it (Heitz-Mayfield and Mombelli, 2014; Froum et al., 2016). The prevention and treatment of PiM gained growing interest.

Mechanical debridement (MD) is recognized as an indispensable and conventional nonsurgical procedure to treat PiM (Figuero et al., 2014; Schwarz et al., 2015) that can result in a clinical improvement and bacterial reduction over 3 months (Máximo et al., 2009). However, a complete resolution of the inflammation and biological complications associated with dental implants in PiM patients cannot be achieved using the MD treatment alone. In order to enhance the efficacy of MD treatment, several adjunctive treatments have been proposed such as antiseptics (Ciancio et al., 1995; Ramberg et at., 2009; Thöne-Mühling et al., 2010), antibiotics (Schenk et al., 1997; Hallström et al., 2012), air abrasive devices (Schwarz et al., 2015), and photodynamic therapy (Takasaki et al., 2009) to achieve a better decontamination of PiM.

Recently, many studies have revealed that the administration of probiotics may have an effect on modulating the composition of oral biofilms and have been investigated in the treatment of periodontal (Vivekananda et al., 2010; Iniesta et al., 2012; Vicario et al., 2013) and peri-implant disease (Flichy-Fernández et al., 2015), caries (Laleman et al., 2014; Gruner et al., 2016), and oral candidiasis (Mendonça et al., 2012; Li et al., 2014). Probiotics have been defined by the World Health Organization in 2001 as “live microorganisms which, when administered as probiotics in adequate amounts, confer a health benefit to the host.” This benefit is obtained through the prevention of the adhesion of endogenous and exogenous pathogens, providing nutrients and cofactors, improving the intestinal barrier integrity, and interacting positively with the intestinal immune system and cell proliferation (Devine and Marsh, 2009). Studies on animal models revealed that pathogenic anaerobic bacteria play a vital role in the etiopathogenesis of periodontitis (Kebschull and Papapanou, 2011). Some periodontopathogens such as *Aggregatibacter actinomycetemcomitans*, *Prevotella intermedia*, *Porphyromonas gingivalis*, *Fusobacterium nucleatum*, and *Tannerella Forsyth*ia are not only significant etiopathogenic factors of the periodontal disease (Colombo et al., 2006) but also commonly related to peri-implantitis (Augthun and Conrads, 1997; Lang N. P. et al., 2011). Oral lactobacilli capable of H_2_O_2_ production inhabit the periodontal pockets in periodontitis patients, with a significantly higher frequency in the moderate form of the disease, as compared to the severe form, and they may prevent the progress to chronic periodontitis, especially by restricting the secretory activity of Th17 cells and growth of periodontopathogens (Szkaradkiewicz and Stopa, 2008; Szkaradkiewicz et al., 2011). Moreover, the application of oral treatment in form of tablets containing the probiotic strain *Lactobacillus reuteri* induces a significant reduction of proinflammatory cytokine (TNF-α, IL-1β and IL-17) response and improves the clinical parameters (SBI, PPD, and CAL) in most patients with periodontitis (Szkaradkiewicz et al., 2014). These proinflammatory cytokines in periodontal pockets play a peculiar role in the induction and development of local inflammatory response, which may determine the clinical form of the periodontal disease (Santos et al., 2010). *Lactobacillus reuteri* and *Lactobacillus salivarius* are the most common probiotics used in clinical practice (Meurman and Stamatova, 2007) known as suppressors of both cariogenic (Nikawa et al., 2004) and periodontal pathogens (Teughels et al., 2013; Tekce et al., 2015). Recently, some clinical studies have focused their attention on the additive effect of probiotics on PiM (Hardar et al., 2016; Galofré et al., 2018; Marta et al., 2019; Fawaz et al., 2019). However, some outcomes in these studies were controversial. Thus, a focused analysis of the adjunctive therapeutic effects of probiotics in the treatment of PiM is needed.

Therefore, the objective of this systematic review and meta-analysis was to investigate the efficacy of the probiotic therapy combined with MD compared with MD alone or MD + placebo in patients with PiM.

## Methods

### Protocol

This systematic review was registered in PROSPERO (CRD42020213625) and conducted in accordance with the recommendations of the Preferred Reporting Items for Systematic Reviews and the principles of PRISMA statement (Moher et al., 2009).

### Focused Question

The present systematic review addressed the following focused question that was structured according to the PICO format: “What is the clinical and microbial efficacy of probiotic therapy additional to MD in patients suffering from PiM when compared with MD alone or MD + placebo?” The patients considered in this systematic review were patients diagnosed with PiM based on case definition used in the publications. The intervention considered in this review was a probiotic therapy additional to a nonsurgical treatment such as MD, representing the experimental group. The experimental group was compared with MD + placebo or MD alone. The considered outcomes were changes in peri-implant mucosal inflammation, such as probing depth (PD), bleeding on probing (BOP), plaque index (PI), and microorganism load and species.

### Search Strategy

A critical review of the literature was performed to select relevant published articles. The following databases such as MEDLINE, Science Direct databases, and Cochrane Central Register of Controlled Trials were used from their inception until November 10th, 2019. Additionally, several journals were manually searched from the following journals: Clinical Oral Implants Research; Clinical Implant Dentistry and Related Research; International Journal of Oral and Maxillofacial Implants; Journal of Periodontology; Journal of Clinical Periodontology. Furthermore, potential review articles and bibliographic references of the included articles were analyzed. When needed, the corresponding authors were contacted to ask to provide missing data or information. The gray literature was consulted using the database System for Information on Gray literature in Europe (http://www.opengrey.eu). A commercially available software (Endnote X7, Thomson, London, United Kingdom) was used for electronic title management. Screening and assessment of potential articles was performed independently by two reviewers blinded to the study (RZ and FJ). Any disagreement between the two reviewers during the first and second stage of the study selection was resolved by discussion.

Keywords from the Medical Subject Headings (MeSH) identified by an asterisk symbol (*) and free text terms were the following: Intervention or Therapy or Treatment or Mechanical debridement or MD Professionally administered plaque removal or PARR or nonsurgical periodontal therapy or nonsurgical therapy or Periodontal treatment or Periodontal Therapy and Probiotic or Probiotic* or Probiotic therapy or Probiotic effect or Probiotic treatment and Peri-implant diseases or Peri-implant mucositis or Mucositis* or Peri-implant.

### Study Inclusion and Exclusion Criteria

During the first stage of the study selection, the titles and abstracts were screened and evaluated. A study was considered eligible for inclusion if it met the following criteria: 1. Randomized controlled clinical trial (RCT, parallel group design) or randomized cross-over study (placebo-controlled) in humans; 2. Evaluation of the results of the treatment performed on PiM patients; 3. Comparison of MD + probiotic *vs.* MD + placebo or MD alone; 4. Data on the clinical changes due to peri-implant mucosal inflammation (i.e., PD, BOP, PI) or microbial outcome after the treatment.

At the second stage of the selection, the full-text articles acquired in the first stage were identified according to the following exclusion criteria: 1. Inclusion of less than five patients; 2. A follow-up assessment less than 6 weeks; 3. Inadequate case definition; 4. Patients who received a surgical treatment; 5. Lack of data on the clinical changes due to PiM inflammation; 6. *In vitro* and animal studies, letters to the editor, opinion articles, review articles, interviews, and monographs.

### Risk of Bias (Quality) Assessment

Two reviewers (RZ and FJ) independently assessed the risk of bias for all the selected RCTs according to the Cochrane Handbook for Systematic Reviews of Interventions (Cochrane Collaboration, 2019). Each criterion was classified as “high risk of bias” (high), “low risk of bias” (low), or “unclear” (?) risk of bias. In this systematic review, five domains (randomization, allocation concealment, participants and professionals blinded to the study, blinding of outcome assessment, and other bias) were chosen as the key domains to evaluate the quality of the studies. Both the reviewers discussed and resolved any disagreements.

### Data Synthesis

Data extraction from the included articles into predesigned data collection template on Microsoft Excel was performed by two reviewers (RZ and FJ) blinded to the study: 1) study identification: first author’s name, year of publication, journal’s name, and country; 2) study design (RCTs); 3) population (subjects): sample size, mean, and age range in years; 4) PiM diagnostic criteria; 5) intervention: details of probiotic administration including dose, frequency, any pretreatment (mechanical or chemical disinfection), and vehicle; 6) smoking habits; and 7) primary and secondary outcomes and observation period. Any discrepancies were resolved by discussion with a third examiner (HHM).

### Data Items

As regards data analysis, the change in PD after treatment was defined as the primary outcome. Secondary outcomes included the absence of BOP, reduction of full mouth PI and BOP, and changes in microorganism number and species.

### Analysis Method

The heterogeneity between the included RCTs was tested and evaluated through Q and I^2^ test. When a *p* value of Q statistic was <0.1, it was defined as an indicator of heterogeneity. The threshold for the interpretation of I^2^ values was also used to estimate the heterogeneity as follows: 0–30% (low heterogeneity), 30–60% (moderate heterogeneity), and >60% (substantial heterogeneity). Differences between MD + probiotic and MD alone or MD + placebo group were expressed as weighted mean differences (WMD) and 95% confidence interval (CI) for continuous outcomes and odds ratio (OR) and 95% CI for dichotomous outcomes, using the random effect models. As regards continuous data, the mean difference and standard error of each study were collected. If data were not reported as mean difference, the mean difference was calculated and the standard deviation was estimated using the r_d_ = sqrt (r_1_^2^/n_1_ + r_2_^2^/n_2_) formula. The meta-analysis was performed using the Review Manager software (RevMan, version 5.3 for Windows).

## Results

### Study Selection

**FIGURE 1:**
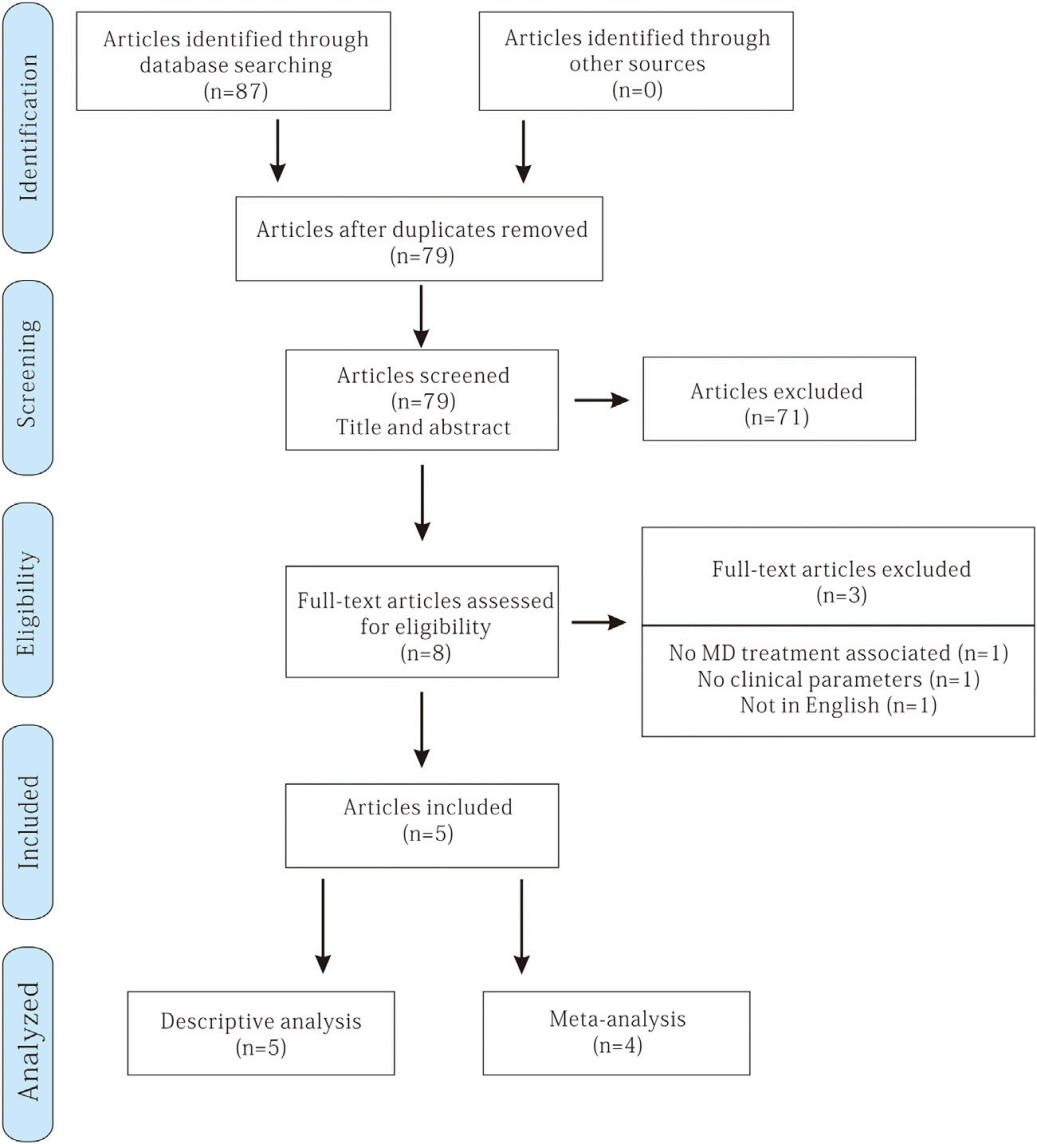
Flow chart of the literature search and inclusion criteria.

A total of 87 potentially relevant titles and abstracts were identified through the electronic and manual search. Among them, 79 articles were excluded based on the title and abstract after removing the duplicates. Therefore, eight remaining articles were assessed for complete evaluation, but among them, three were excluded because they did not fulfill the inclusion criteria. Finally, five studies met the inclusion criteria and were included in this systematic review (Hardar et al., 2016; Mongardini et al., 2017; Galofré et al., 2018; Marta et al., 2019; Fawaz et al., 2019) (Figure 1).

### Excluded Studies

The reasons for excluding specific studies are summarized in Table 1. One study reported probiotic therapy without nonsurgical mechanical therapy (Flichy-Fernández et al., 2015), and one study lacked the data of clinical examination of the implant inflammation (Lauritano et al., 2019). The other study was excluded because it was written in Russian (Ahmedbeyli et al., 2019).

**TABLE 1:** Excluded clinical studies at the second stage of selection and the reason for exclusio

Publication	Reason for exclusion
Flichy-Fernández et al. (2015)	Without non-surgical mechanical therapy in two groups
Lauritano et al. (2019)	Lack of parameters of clinical examination and unclear definition of PiM
Ahmedbeyli et al. (2019)	Published in Russian

### Study Characteristics

The characteristics of the five included studies are reported in Table 2. All of them were RCTs and published in the English language between 2015 and 2019, four with a parallel design (Hardar et al., 2016; Galofré et al., 2018; Marta et al., 2019; Fawaz et al., 2019) and one with a cross-over design (Mongardini et al., 2017). All the included studies were conducted at a single center and were designed for comparison between groups. Three of them included up to 40 patients (Hardar et al., 2016; Marta et al., 2019; Fawaz et al., 2019) and Galofré et al. (2018) and Mongardini et al. (2017) included up to 20 patients. The follow-up period ranged from 6 (Mongardini et al., 2017) to 26 weeks (Hardar et al., 2016). Three studies (Hardar et al., 2016; Galofré et al., 2018; Marta et al., 2019) performed an intermediate evaluation at 3 months.

**TABLE 2:** Characteristics of the included studies.

Study Journal Region	Type	Number of implants	Clinical parameters	Subjects M/F Age	Peri-implant mucositis definition	Treatment	Probiotic Administration	Smoking	Follow-up	Mean (SD) Outcome
Hardar et al. (2016) Acta Odontol Scand Sweden	RCT Placebo double-blind Parallel	one or more per subject	PI, BOP, PPD, presence of pus, Microbiological parameters	49 18/31 53.7/63.3	PD ≥4mm + BOP and/or pus+ CBL < 2mm	With OHI MD + placebo (control) MD+ probiotic (test)	A droplet of oil containing Lactobacillus reuteri applied around implant. Lactobacillus reuteri 2 lozenges per day for 3 months started at onset of initial therapy	29%/8%	26 weeks (days 7,14,28,84,182)	Test: Presence of PI (%): 26 (BL) to 9 (3 months, Implant Level) Presence of BOP(%): 54 (BL) to 11 (3 months, Implant Level) (Sign.) Presence of pus (%): 13 (BL) to 2 (3 months, Implant Level) PD: 4.3 (SD:1.1) (BL) to 3.7 (SD:1.2) (3 months, Implant Level) Control Presence of PI(%): 32 (BL) to 10 (3 months, Implant Level) Presence of BOP(%): 58 (BL) to 18 (3 months, Implant Level) (Sign.) Presence of pus (%):5 (BL) to 0 (3 months, Implant Level) PD: 4.0 (SD:1.4) (BL) to 3.4 (SD:1.4) (3 months, Implant Level)
Mongardini et al. (2017) *J Clin Periodontol* Italy	RCT Placebo Cross-over	20	Modified PI number of BOP+ sites per implant unit	20 9/11 57	BOP, plaque accumulation+ CBL < 2mm	With OHI MD+PDT +placebo (control) MD+PDT +probiotic (test)	Probiotic mixture was delivered to the peri-implant sulcus; one probiotic tablet containing Lactobacillus taken per day for 14 days at onset of initial therapy	No or former smokers	6 weeks (days14, 42)	Test: mPI: 1.2 (SD:0.49) (BL) to 0 (SD:0.12) (6 weeks, Implant Level) (Sign.) number of BoP+ sites per implant unit : 4 (SD:2.2) (BL) to 2 (SD:1.48) (6 weeks, Implant Level) (Sign.) Control: mPI: 1.42 (SD:0.76) (BL) to 0.17 (SD:0.24) (6weeks, Implant Level) (Sign.) number of BoP+ sites per implant gunit: 3.5 (SD:2.9) (BL) to 2 (SD:2.2) (6 weeks, Implant Level) (Sign.)
Galofré et al. (2018) J Periodont Res Spain	RCT Placebo Parallel	22	PI, BOP, PPD Microbiological parameters	22 64/36 61.5/60.0	BOP and/or pus + no evidence of radiographic bone loss .	Without OHI MD+ placebo (control) MD + probiotic (test)	Lactobacillus reuteri 1 lozenges per day for 30 days started at onset of initial therapy	No	3 months (days 30.90)	Test: PI:0.41(SD:0.21) (BL) to 0.25 (SD:0.10) (3 months, Subject Level)(Sign) Presence of PI(%): 72.7 (BL) to 27.3 (3 months, Implant Level) (Sign.) BOP: 0.61 (SD:0.27) (BL) to 0.29 (SD:0.09) (3 months, Subject Level) (Sign.) Presence of BOP(%): 100(BL) to 54.5 (3 months, Implant Level) PPD: 3.84 (SD:0.55) (BL) to 3.35 (SD:0.76) (3 months, Implant Level) (Sign.) Control: PI:0.39 (SD:0.10) (BL) to 0.29 (SD:0.10) (3 months, Subject Level) (Sign.) PI(%): 54.5 (BL) to 63.6 (3 months, Implant Level) BOP: 0.42 (SD:0.18) (BL) to 0.35 (SD:0.22) (3 months, Subject Level) BOP(%): 100 (BL) to 90.9 (3 months, Implant Level) PPD: 3.82 (SD:0.64) (BL) to 3.66 (SD:0.62) (3 months, Implant Level) (Sign.)
Marta et al. (2019) Clinical Oral Investigation Spain	RCT Placebo Parallel	50	PI, BOP, PPD, Microbiological parameters	50 42/58 55.96/61.16	BOP+ no radiographic signs of bone loss	With OHI+ Chlorhexidine (day0-day 15) MD + placebo (control) MD+ probiotic (test)	Lactobacillus reuteri 1 lozenges per day for 30 days from day 15-day 45	No	3 months (days 90)	Test: PI:0.31(SD:0.13) (BL) to 0.24 (SD:0.09) (4 months, Subject Level) (Sign) Presence of PI (%): 72 (BL) to 24(3 months, Implant Level) (Sign.) BOP: 0.20 (SD:0.13) (BL) to 0.12 (SD:0.06) (4 months, Subject Level) (Sign.) Presence of BOP (%): 100(BL) to 64 (4 months, Implant Level) PD: 3.10 (SD:0.74) (BL) to 2.88 (SD:0.62) (4 months, Implant Level) (Sign.) Control: PI:0.33 (SD:0.12) (BL) to 0.26 (SD:0.10) (4 months, Subject Level) (Sign.) Presence of PI (%): 54.5 (BL) to 63.6 (4 months, Implant Level) (Sign.) BOP: 0.20 (SD:0.11) (BL) to 0.15 (SD:0.08) (4 months, Subject Level) Presence of BOP (%): 100 (BL) to 60 (4 months, Implant Level) PD: 3.32 (SD:0.65) (BL) to 2.98 (SD:0.60) (4 months, Implant Level) (Sign.)
Fawaz et al. (2019) Clin Implant Relat Res Saudi Arabia	RCT Placebo Parallel	40	PI, BOP, PPD	80 80/0 36.1	BOP + PD ≥3mm at 30% sites + CBL < 2mm	With OHI + Chlorhexidine (day0-day 14) MD alone (control) MD+ probiotic (test)	Lactobacillus reuteri 2 lozenges per day for 3 weeks Started at onset of initial therapy	50.0%	6 months (days 90, 180)	Test: Presence of PI (%): 48.3 (SD:6.2) (BL) to 10.2 (SD:5.2) (3 months, Implant Level) (Sign.) Presence of BOP (%):50.5 (SD:7.3) (BL) to 19.2 (SD:4.1) (3 months, Implant Level) (Sign.) PD:3.5 (SD:0.2) (BL) to 0.9 (SD:0.3) (6 months, Implant Level) (Sign.) Control: Presence of PI(%): 48.3 (SD:6.2) (BL) to 24.2(SD:5.3) (3 months, Implant Level) Presence of BOP(%):50.5 (SD:7.3) (BL) to 28.3 (SD:6.1) (3 months, Implant Level) PD:3.5 (SD:0.2) (BL) to 3.3 (SD:0.5) (6 months, Implant Level)

BL, baseline; BOP, bleeding on probing; CBL, crestal bone loss; MD, mechanical debridement; M/F, Male/Female; OHI, oral hygiene instructions; PI, Plaque Index; PPD, pocket probing depth; RCT, randomized clinical trial.

### Treatment Modalities

Nonsurgical mechanical therapy was always performed using ultrasonic scalers and manual instruments. Oral hygiene instructions were provided in all the studies except one (Galofré et al., 2017). All studies used the probiotic strain *Lactobacillus*. The types of administration were as follows: three trials (Galofré et al., 2018; Fawaz et al., 2019; Marta et al., 2019) used tablets (containing *L. reuteri* DSM 17938 and *L. reuteri* ATCC PTA 5289). Two trials (Hardar et al., 2016; Mongardini et al., 2017) used both tablets and probiotic mixture around the implant. The probiotic supplementation period in these studies was 2 weeks (Mongardini et al., 2017), 3 weeks (Fawaz et al., 2019), 30 days (Galofré et al., 2018; Marta et al., 2019), and 3 months (Hardar et al., 2016).

Three studies included participants who were only nonsmokers or former smokers (Mongardini et al., 2017; Galofré et al., 2018; Marta et al., 2019). Two studies (Hardar et al., 2016; Fawaz et al., 2019) included both nonsmokers and smokers and reported the population of each type.

### Risk of Bias

Every effort was made to retrieve from the authors all missing data in the included studies according to the advice in Section 16.1.2 of the Cochrane Handbook. However, although the authors were contacted by electronic mail (Hardar et al., 2016; Mongardini et al., 2017; Fawaz et al., 2019) in order to obtain information not reported in the articles, no answer was received.

**FIGURE 2:**
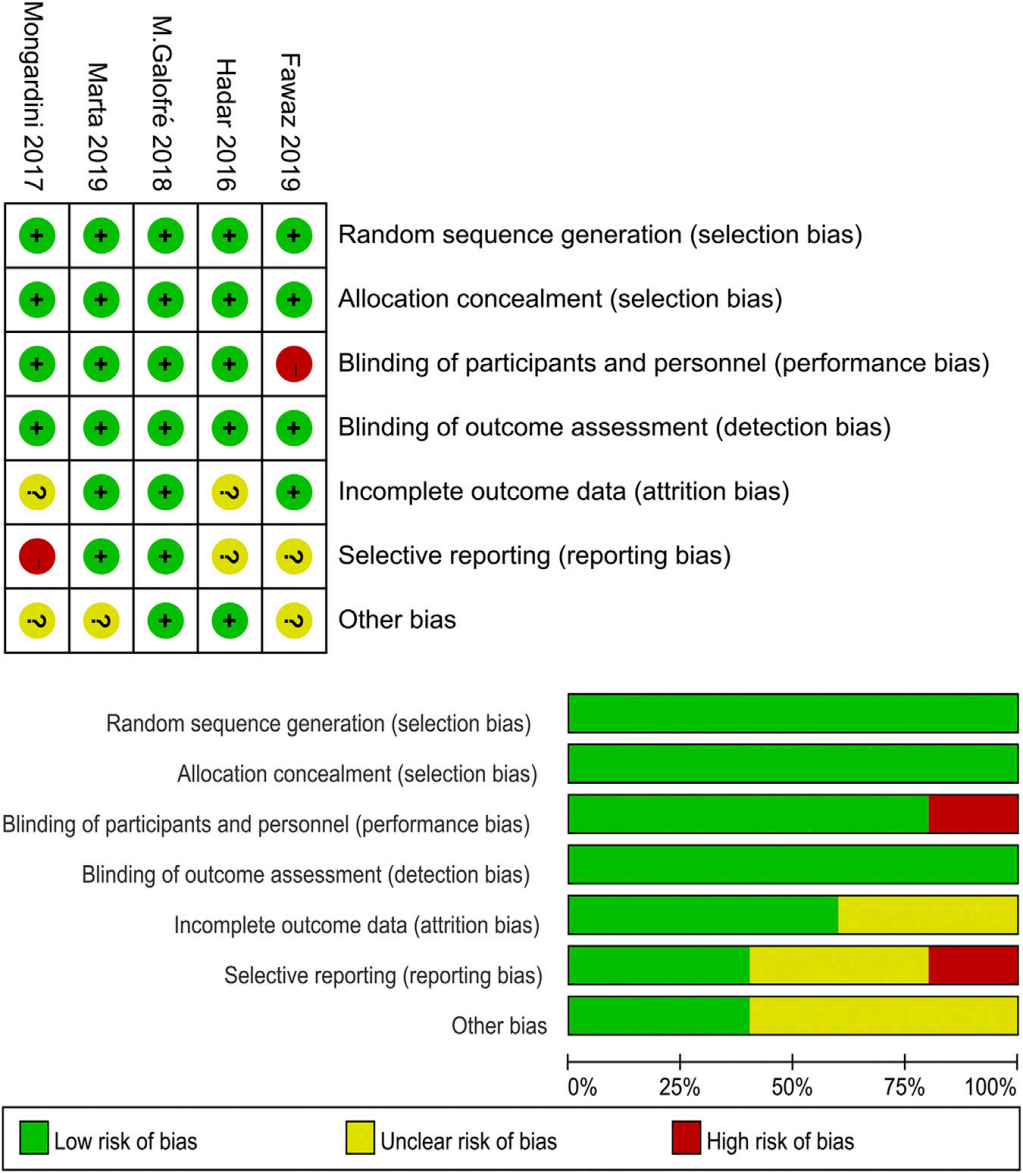
Quality assessment of the selected studies (The Cochrane Collaboration tool for assessing risk of bias).

Two studies (Mongardini et al., 2017; Fawaz et al., 2019) were considered as having a “high” risk of bias. The study of Mongardini et al. (2017) was an RCT with a cross-over design, and they did not use paired test for the statistical analysis of the data between groups, which lead to a lack of data. Fawaz et al. (2019) did not use placebo in the control group; thus, the blinding to the study by the participants cannot be verified. More information about the risk of bias assessment is shown in Figure 2.

### Study Outcomes

#### PD Reduction

At 3 months, two studies (Galofré et al., 2018; Fawaz et al., 2019) reported a significant difference on implant PD reduction (*p* < 0.5) in favor of the nonsurgical MD + probiotic group when compared with the nonsurgical MD + placebo. On the contrary, the study of Hardar et al. (2016) and Marta et al. (2019) did not highlight any difference between them. At the end of the observation period, the mean PD reduction in the nonsurgical MD + probiotic group ranged from 0.24 (±0.48) (Marta et al., 2019) to 0.75 (±0.53) mm (Fawaz et al., 2019), while this reduction ranged from 0.15 (±0.36) (Galofré et al., 2018) to 0.5 (±0.55) (Fawaz et al., 2019) in the MD + placebo.

#### BOP and PI Changes

In two studies (Galofré et al., 2018; Marta et al., 2019), the results of the treatment were recorded according to the absence or presence of BOP and PI considering the patient as a unit and reported the improvement of BOP and PI in both the experimental and placebo group without significant difference among them. Hardar et al. (2016) recorded BOP and PI at four sites of the selected implants, considering the implant as a unit, and they also reported a similar outcome. In the study of Fawaz et al. (2019), as regards never-smokers in the PiM group, the mean score of BOP and PI was significantly higher among patients that underwent MD alone compared with patients who underwent MD + probiotic at 3-months of follow-up (*p* < 0.05).

#### Microbiological Outcomes

Three studies (Hardar et al., 2016; Galofré et al., 2018; Marta et al., 2019) performed the collection of the biological material in the deepest peri-implant pockets (Table 2). The samples were collected using sterile paper strips for 15 s (Galofré et al., 2018; Marta et al., 2019) and 20 s (Hardar et al., 2016) in the peri-implant pocket. Only Hardar et al. (2016) reported the time between the collection and processing of the samples. Colony-forming units (CFU) were used to estimate the change in microbial load of all types of microorganisms in all studies (Table 3).

**TABLE 3:** Microbiological methods of the selected studies.

Study Journal Region	Instrument collection	Load implant collection	Time	Transport media/Processing	Technique
Galofré et al. (2018)	Sterile paper tips	Deepest PPD	10–15 s	In a microfuge tube/WD	Quantiative (CFU)
Hardar et al. (2016)	Paper points	Deepest PPD	20 s	In an Eppendorf tube/30min	Quantiative (CFU)
Marta et al. (2019)	Sterile paper tips	Deepest PPD	15 s	In an Eppendorf tube/WD	Quantiative (CFU)

CFU, colony-forming units; PPD, pocket probing depths; WD, without data.

**TABLE 4:** Microbiological results for selected studies.

Author/year	Microorganisms											
	Total microorganisms		P. gingivalis		A. actinomycetemcomitans		P. intermedia		F. nucleatum		T. forsythia	
	Time point	p < 0.05	R	p < 0.05	R	p < 0.05	R	p < 0.05	R	p < 0.05	R	p < 0.05
Galofré et al. (2018)	AT	Y	N	—	—	Y	N	Y	N	Y	N	
	1 month	N	Y	N	—	—	Y	N	Y	N	Y	N
	3 months	N	Y	Y	—	—	Y	N	Y	N	Y	N
Hardar et al. (2016)	AT	N	Y	N	Y	N	Y	N	Y	N	Y	N
	1 month	N	Y	N	Y	N	Y	N	Y	N	Y	N
	3 months	N	Y	N	Y	N	Y	N	Y	N	Y	N
Marta et al. (2019)	AT	N	Y	N	—	—	Y	N	Y	N	Y	N
	1 month	N	Y	N	—	—	Y	N	Y	N	Y	N
	4 months	Y	Y	N	—	—	Y	N	Y	Y	Y	N

AT, after treatment; N, no; R, results; Y, yes.

#### Meta-Analysis

A meta-analysis was conducted on studies which reported a similar assessment of PD, PI, BOP, or microorganisms.

Based on four studies (Hardar et al., 2016; Galofré et al., 2018; Marta et al., 2019; Fawaz et al., 2019), the WMD in PD reduction between experimental and control group was −0.12 mm [95% CI (−0.38, 0.14), *p* = 0.38], not favoring the additional probiotic therapy (*p* value for heterogeneity: 0.15, I^2^ = 43% = moderate heterogeneity) (Figure 2).

**FIGURE 3:**
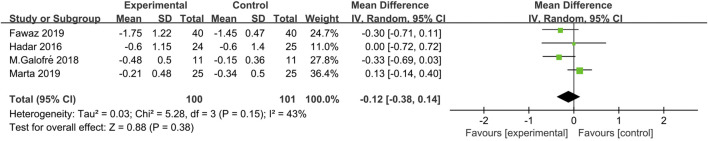
Forest plot of PD reduction at implant level at 3 months.

In two studies (Galofré et al., 2018; Marta et al., 2019), the reduction of full mouth PI and full mouth BOP and absence of BOP at the implant level were assessed. Interstudy heterogeneity appeared significant regarding full mouth BOP reduction (*p* < 0.1, I^2^ = 79%) and absence of BOP at implant (*p* < 0.1, I^2^ = 66%). The meta-analysis failed to show a significant full mouth BOP reduction (*p* = 0.31) and absence of BOP at the implant site (*p* = 0.52) between MD + probiotic and placebo (Figure 3). WMD in full mouth PI reduction between experimental and placebo group was −0.00 [95% CI (−0.02, 0.02), *p* = 0.85] also not favoring the additional probiotic therapy (*p* value for heterogeneity: 0.23, I^2^ = 30% = low heterogeneity) (Figure 3).

**FIGURE 4:**
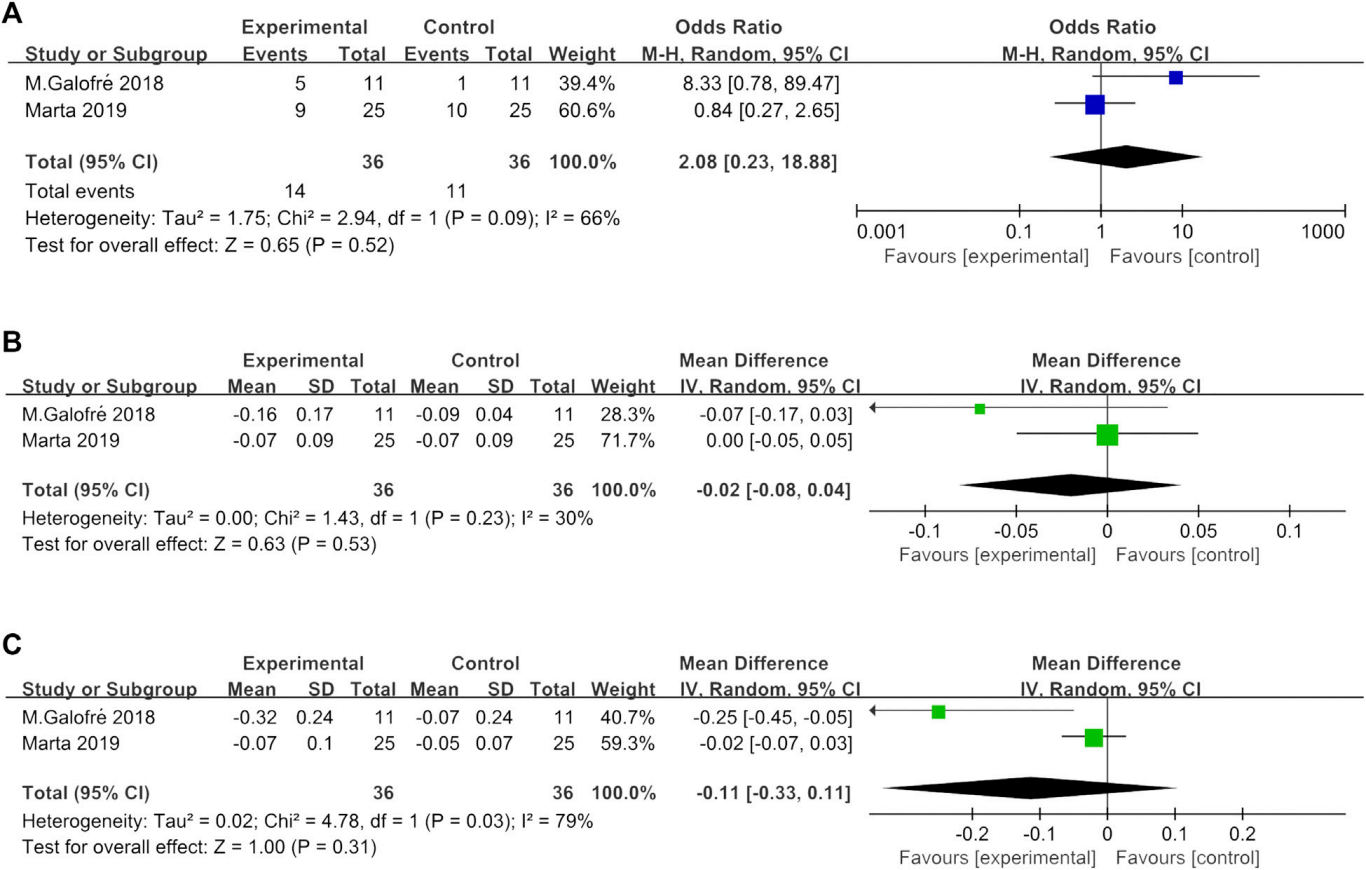
**(A)** Forest plot of BOP absence at implant level at 3 months. **(B)** Forest plot of the overall FMPI reduction at 3 months. **(C)** Forest plot of the overall FMBOP reduction at 3 months.

Two studies (Galofré et al., 2018; Marta et al., 2019) showed the number of CFU of the total bacterial load associated with *P. gingivalis*, *P. intermedia*, *T. forsythia*, and *F. nucleatum* counts (Figure 4). The heterogeneity between trials was high, except for *T. forsythia* (*p* = 0.24, I^2^ = 27% = low heterogeneity). The meta-analysis of microbiological changes was not in favor of the additional antiseptic therapy compared to MD alone.

**FIGURE 5:**
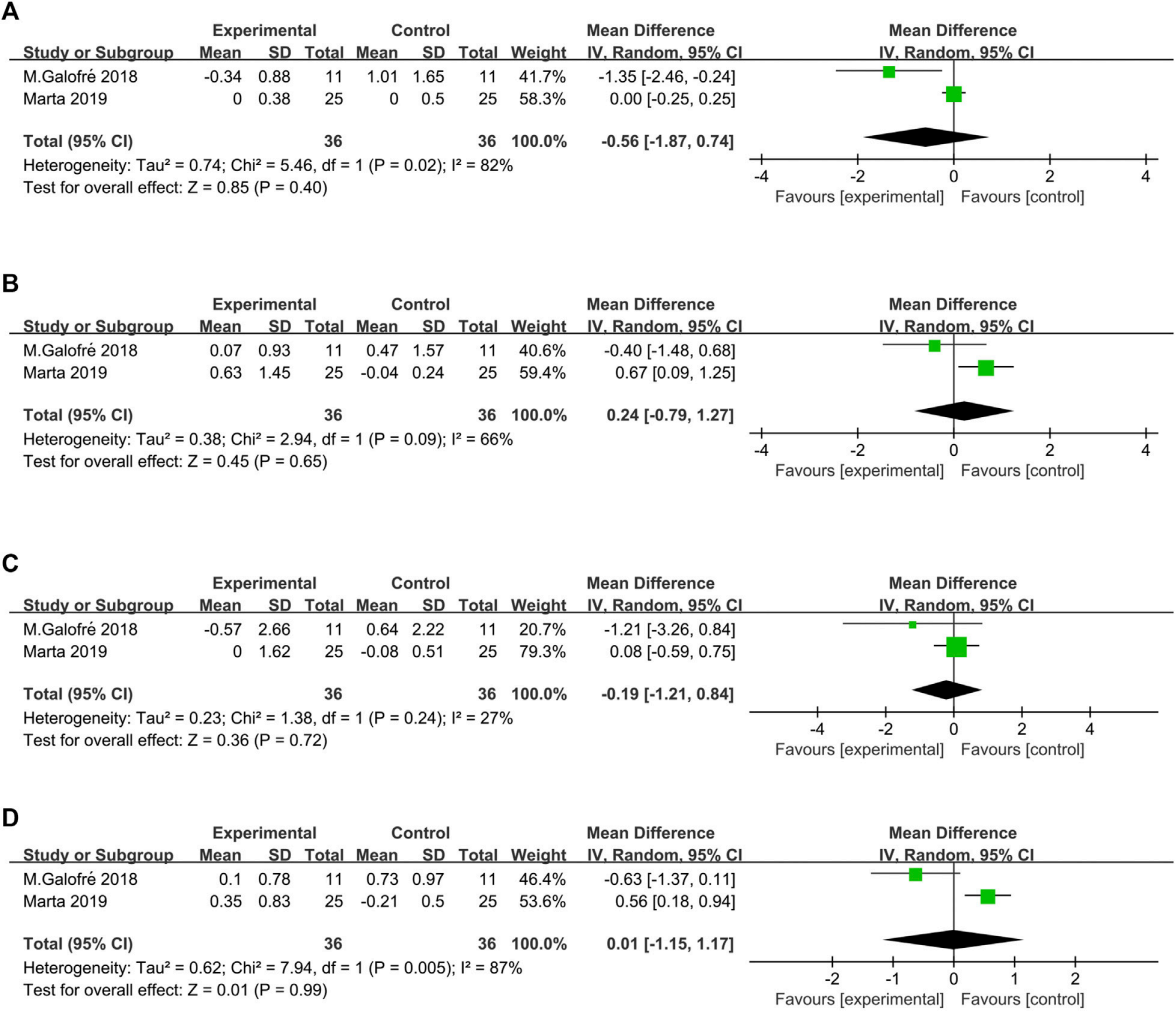
Forest plot of the microorganism load regarding the established subgroups: **(A)** *P. gingivalis*; **(B)** *P. intermedia*; **(C)** *T. forsythia*; **(D)** *F. nucleatum*.

## Discussion

With the overuse of antibiotics and the increasing problem of bacterial resistance, probiotic treatment has been widely applied to cure and/or prevent many infectious diseases as an alternative adjunctive treatment to encourage microflora balance. In the healthy mouth environment, human oral cavity is a complicated and relatively balanced microecosystem with thousands of different types of bacteria. The shift or disorder in the bacterial community, such as the reduction of symbiont microbiota and the increase of pathogenic microbiota, may often predict various local infections and inflammations including gingival, periodontal, and endodontic diseases (Moore et al., 1982; Sassone et al., 2012). *Lactobacillus* is a widely used probiotic and some studies showed that adequate administration of *L. reuteri* can result in a beneficial effect in the maintenance of the ecological balance in the intestinal tract as well as in the oral cavity (Reuter, 2001; Vivekananda et al., 2010).

The present systematic review and meta-analysis was performed to evaluate the scientific evidence on the following focused question: What is the clinical and microbial efficacy of probiotic therapy additional to MD in patients suffering from PiM when compared with MD alone or MD + placebo, from the clinical and microbiological point of view? However, the present evidence available did not support any significant difference to allow statements regarding the efficacy of probiotics.

In terms of the primary outcome, the magnitude of the reduction in PD varied among the included studies. Three studies revealed a decrease in PD that was generally less than 1 mm (Hardar et al., 2016; Galofré et al., 2018; Marta et al., 2019) and one study reported a PD reduction to 1 mm in both test and control group. Data synthesis of the respective RCTs revealed that WMD in PD reduction was not in favor of the additional probiotic therapy over MD alone [SD = −0.12 mm 95% CI (−0.38, 0.14), *p* = 0.38] at 3 months. As regards the secondary outcomes (such as full mouth PI, full mouth BOP, absence of BOP, and microbiological load), the meta-analysis evaluated the difference based on the results of two included studies, also lacking significant differences between experimental and probiotic group. According to previous studies, the key parameter in the diagnosis of PiM is the bleeding on gentle probing (<0.25 N) (Lang N. et al., 2011). Thus, the absence of BOP was an indication of the resolution of the peri-implant mucosal inflammation, suggesting the endpoint of the following nonsurgical treatment. Since only two studies reported the absence of BOP at the implant level and the data were analyzed in the meta-analysis, the conclusion was that the adjunctive probiotic therapy could not improve the peri-implant mucosal inflammation. Three studies also reported the microbiological changes after therapy (Hardar et al., 2016; Galofré et al., 2018; Marta et al., 2019). One article (Hardar et al., 2016) was excluded from the meta-analysis because it presented only the prevalence of the selected bacterial strains (>10^4^ CFU) without the amount related to the microorganism reduction. In consideration of the high heterogeneity of the parameters evaluated and the small number of eligible studies included in this meta-analysis, these results of microbial load reduction represented preliminary evidence. No significant reduction in *P. gingivalis, P. intermedia*, *T. forsythia*, and *Fusobacterium nucleatum* CFU counts was observed. Taken together, the present findings from all data indicated no additional clinical or microbiological benefit due to the administration of *Lactobacillus strains* as the additional therapy to MD when compared with MD with placebo or MD alone.

Regarding the treatment of PiM, several additional therapies (such as antiseptic and systemic antibiotic therapy) to MD have already been evaluated in order to gain better control of the progression of the disease. A meta-analysis with seven including studies evaluating the use of adjunctive measures (antiseptic and systemic antibiotic therapy, air abrasive device use) to remove plaque in PiM patients (Schwarz et al., 2015) reported a PD reduction of −0.056 mm (−0.27, 0.16 mm), which was similar to our results. The same author performed another meta-analysis (Schwarz et al., 2015) to compare the efficacy of nonsurgical (referring to PiM and peri-implantitis) and surgical (referring to peri-implantitis) treatment with alternative or additional therapy on changes of inflammation compared with conventional nonsurgical and surgical treatments alone. The systematic review concluded that MD alone was effective in the management of PiM, while the alternative or additional therapy may improve the efficacy over/of conventional nonsurgical treatments at peri-implantitis sites. Therefore, our evidence and the one of Schwarz et al. both support that certain adjunctive therapies cannot bring evident clinical benefits compared to MD alone in the treatment of PiM.

Considering that this meta-analysis is firstly performed on this topic (additional probiotic treatment), it is difficult to compare the results of the present meta-analysis with previous systematic reviews. In the evaluation of the systematic review design, the assessment of quality and risk of bias for all included studies is very crucial. In a systematic review, the included studies inevitably have some differences. Therefore, some important issues should be taken into consideration when analyzing the results. At first, since all the included studies used the same probiotic (*Lactobacillus strains*), the conclusion of this systematic review could not be generalized to other types of probiotics. Moreover, they demonstrated some variability in the type of probiotic used, dose, and method of administration. For instance, three studies (Mongardini et al., 2017; Galofré et al., 2018; Marta et al., 2019) reported that the participants were treated with one probiotic lozenge per day; in the other two studies (Hardar et al., 2016; Fawaz et al., 2019) they were treated with two lozenges per day. Second, the included studies used different oral hygiene programs. Only one study (Galofré et al., 2018) did not give patients specific oral hygiene instruction during the trial. Two studies used chlorhexidine at the beginning of the treatment. Third, since smoking has been confirmed as a risk factor of PiM or peri-implantitis, only three studies included patients who were nonsmoker or former smoker, while one study recruited both smoker and nonsmoker (Hardar et al., 2016). Fourth, some included studies reported the outcomes with different measuring method, such as the information on plaque control, BOP, or microbiological load. In order to find more useful data, Hardar and Fawaz were contacted, but they did not reply. Furthermore, it should be noticed that a short follow-up period, ranging between 6 and 26 weeks, in these selected articles was considered when interpreting the presented results. Accordingly, the long-term effect of an additional probiotic treatment is unknown and needs further investigation. Finally, because of the high heterogeneity, the limited available data of the included studies, and the small size of the studies analyzed in our review, the quality of the evidence might be decreased, and the impact of the conclusions of this meta-analysis could be reduced.

## Conclusion

The findings of this systematic review and meta-analysis suggested that the additional use of probiotics (*Lactobacillus* strains) did not improve the efficacy of MD in PiM treatment in clinical and microbial outcomes at short-term. This treatment protocol showed similar results when other additional therapies were used to treat PiM. However, because of the heterogeneity and limited available data of the included studies, well-designed long-term RCTs with large sample sizes are needed.

## Data Availability Statement

The original contributions presented in the study are included in the article/Supplementary Material, further inquiries can be directed to the corresponding author.

## Author Contributions

RZ performed the data collection and data analyses and wrote the manuscript. FJ carried out research design, data collection, and data analyses. HH helped with data analyses. YW helped perform the analysis with constructive discussions. WL contributed to the conception of the study and guidance of manuscript writing.

## Conflict of Interest

This study was supported by grants from the Science and Technology Department of Sichuan Province (No. 2018SZ0232). The authors declare that the research was conducted in the absence of any commercial or financial relationships that could be construed as a potential conflict of interest.
